# Epigenetic modification and therapeutic targets of diabetes mellitus

**DOI:** 10.1042/BSR20202160

**Published:** 2020-09-10

**Authors:** Rajveer Singh, Shivani Chandel, Dhritiman Dey, Arijit Ghosh, Syamal Roy, Velayutham Ravichandiran, Dipanjan Ghosh

**Affiliations:** 1 National Institute of Pharmaceutical Education and Research, Kolkata 164, Manicktala Main Road, Kolkata 700054, India; 2 Department of Chemistry, University of Calcutta, Kolkata 700009, India

**Keywords:** diabetes mellitus, epigenetics, Molecular Targets

## Abstract

The prevalence of diabetes and its related complications are increasing significantly globally. Collected evidence suggested that several genetic and environmental factors contribute to diabetes mellitus. Associated complications such as retinopathy, neuropathy, nephropathy and other cardiovascular complications are a direct result of diabetes. Epigenetic factors include deoxyribonucleic acid (DNA) methylation and histone post-translational modifications. These factors are directly related with pathological factors such as oxidative stress, generation of inflammatory mediators and hyperglycemia. These result in altered gene expression and targets cells in the pathology of diabetes mellitus without specific changes in a DNA sequence. Environmental factors and malnutrition are equally responsible for epigenetic states. Accumulated evidence suggested that environmental stimuli alter the gene expression that result in epigenetic changes in chromatin. Recent studies proposed that epigenetics may include the occurrence of ‘metabolic memory’ found in animal studies. Further study into epigenetic mechanism might give us new vision into the pathogenesis of diabetes mellitus and related complication thus leading to the discovery of new therapeutic targets. In this review, we discuss the possible epigenetic changes and mechanism that happen in diabetes mellitus type 1 and type 2 separately. We highlight the important epigenetic and non-epigenetic therapeutic targets involved in the management of diabetes and associated complications.

## Introduction

Diabetes mellitus is a chronic metabolic disorder connected with several environmental and genetic elements. The incidence of diabetes has increased worldwide with a higher rate that accelerated the various life-threatening complications like diabetes-induced retinopathy, nephropathy, neuropathy and other cardiovascular problems such as hypertension, stroke and atherosclerosis [1–6]. Type 1 diabetes is an autoimmune disorder identified by the destruction of the β-cells of pancreas and impaired secretion of insulin. Type 2 diabetes (T2D) is characterized by insulin resistance in impaired cell functions. Epigenetic factors have a very serious influence on target organs which causes irregular cell transcription such as abnormal growth promotion, improper pro-fibrotic and pro-apoptotic gene expression [6–9]. Epigenetic factors play a very important role in diabetes mellitus, in spite of having no specific definition of epigenetic modification. But it is elaborated as a heritable alteration in gene expression without any changes in deoxyribonucleic acid (DNA) sequence [10]. These modifications can be passed from one organism to its progeny. Epigenetic factor encompasses cytosine methylation, histone modification and non-coding microRNAs (miRNA). These factors influence the gene expression level either independently or collectively [11]. Environmental factors that contribute toward the development of T2D are the suboptimal *in utero* environment, obesity, advanced age and low birth weight [11]. Cytosine is a target of DNA methylation in vertebrates and leads to transcriptional silencing. The transcriptional silencing occurs via recruitment of repressor protein or by binding of a transcription factor that only binds with methylated CG island (such as methyl CpG-binding protein 2 (MeCP2)) [12].

## Epigenetic changes responsible for diabetes mellitus type 1

Type 1 diabetes mellitus (T1D) is an autoimmune disorder resulting from the several interconnected factors such as genetic, epigenetic changes and environmental factors [13]. Globally, the incidence of T1D is increasing dramatically in children up to 15 years old with a range of ∼3 to ∼5% per year [14]. The resulted cases have been reported approximately ∼65000/year in children and teenagers [15–18]. The notable growth of T1D incidence proposed the significance of epigenetic changes and environmental factor in the etiology of T1D [19–22]. Environmental factors involved in T1D includes environmental pollutants, exposure with vitamin D, milk protein consumption and infection with retroviruses [13,22,23]. The chances of increase in the incidence of T1D is due to its inheritability since the percentage of incidence of T1D is ∼7% in sibling but in twins, the percentage is approximately 12–67.7%, which is 0.4% of the total population [12,13]. Actually, Type 1 diabetes is mostly a heritable disorder, mainly occurring in sibling and with 15-year-olds and largest chances in autoimmune susceptible monozygotic twins [8,13]. These factors collectively explain the epigenetic modifications in the expression of the gene and give strong evidence about the complex relationship between environment and genetic factors. The epigenetic modification in altered gene mainly involved DNA hypermethylation, histone deacetylation and miRNA dysregulation [14].

These factors have effects on insulin secretion and Type 1 diabetes risk. Several examples can explain the possible relation between the epigenetic modification and exploring the condition causing the T1D.

### DNA methylation

**Figure 1:**
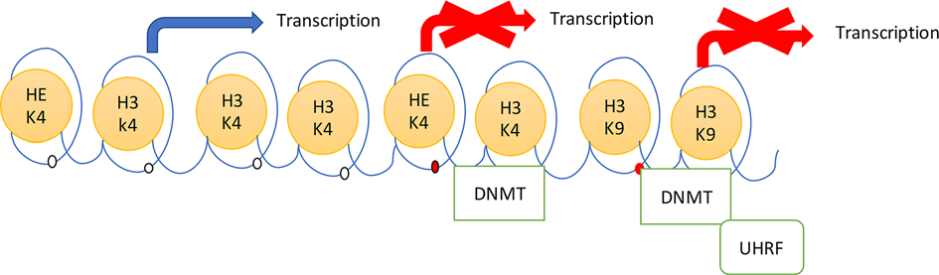
The histone methylation protein attached to N-terminal of H3; in unmethylated form it leads to start the transcription In the presence of DNMT that methylated the H3 of the histone resulted in the *de novo* methylation of DNA. The link of H3K9 with methylation of histone is carried out in the formation of complex of DNMT and UHRF, which resulted in repression of transcription. Abbreviation: UHRF, ubiquitin-like, containing PHD and RING finger domains, 1.

CpG sequence is short stretches of palindromic DNA; present on both strands. 5-Methyl cytosine is a result of methylation of DNA at fifth carbon of cytosine (Figure 1) [15]. DNA-methyl transferase enzymes (DNMTs) are responsible for transfer of methyl group from the donor S-adenosyl methionine to the DNA. In the mammalian genome, majority of CpG dinucleotides are methylated. The ability of DNA methylation to regulate gene expression is determined by gene regulatory region and gene bodies, effects of DNA methylation are dependent on its position in the gene [15]. Methylation in gene body is associated with decreased ability of RNA polymerase transcription of gene. Methylation located in the promoter or first intron (regulatory region) of the gene is important for regulation of transcription of initiation and generally associated with gene silencing. Mechanism of gene silencing can be described by two non-exclusive models: (1) access of transcriptional co-activators to their cognate sequence is blocked by methyl groups, (2) 5-methyl-CpG induces repressive state of chromatin. Weber et al. [16] divided gene promoters into three categories as per their C, G and CpG density.

Studies on promoter DNA methylation on gene expression were done: (1) promoters with low CpG density were common among tissue-specific genes and were found to be methylated without any link to gene activity; (2) promoters with high CpG density were mostly found with housekeeping genes and were non-methylated irrespective of promoter activity; (3) promoters with intermediate CpG density were intermediary methylated and promoter methylation correlated with gene expression [16].

Studies showed that non-methylated CpG are present at the beginning of transcription near to SXY boxes binding transcription factors and methylation in this region results in suppression of transcription [17].

Hypermethylation is commonly related to the gene silencing, and hypomethylation leads to increased gene expression. Various reports are mentioned on distinct methylation level of genes related to the T1D patients [18–20]. The difference in level of methylation between T1D patient and an individual was found to be on four CpG sites present proximally to the insulin gene and encodes pre-proinsulin. This gene has second highest elevated odds ratio for the development the risk of T1D. Hypomethylation of CpG-19, 234 and 135 and hypermethylation of CpG-180 have been associated with T1D risk [21]. The difference in the ratio of circulating methylated and unmethylated insulin genes has been related to the generation of T1D risk. The higher level of methylated DNA produced by β-cell may be used as possible biomarker for destruction of β-cell in T1D [22]. Study on 252 T1D patents and 286 normal individuals revealed that T1D patients had hypermethylation at CpG-373 and 456 [23]. Methylation of CpG island in the lactate dehydrogenase C has been related to the generation of insulin auto-antibodies in children, which leads to elevated T1D risk.

### Histone modification

Histones undertake the post-translational modifications on particular residues of amino acids at N-terminal of the histone protein. The importance of the modification depends upon type of modification (methylation, acetylation, phosphorylation and ubiquitination), degree of modification (mono-, di- and tri-methylation) and on the position of the modification. The defined modification is exactly recognized by the remodeling of chromatin protein which eventually modifies the degree of chromatin condensation. The level of gene expression based upon the territory of the modification (such as gene body, promoter and CpG island), combination and pattern of modification [24]. Histone acetylation at H3 and H4 is the principal marker of activation for the transcription, due to the negative charge of these proteins and which put aside the DNA negative charged molecule. The acetylation process is reversible in nature, it happens at lysine residue of the histone protein. Whole pathway is managed by the histone-acetyltransferases (HATs) and histone deacetylases (HDACs) that has significance as the transcription co-activator and co-repressor. The deletion of the acetyl group leads to the condensation of the histone and repression of transcription. The lower expression of the acetylation leads to facilitate the further modification of histone protein and methylation of the DNA. The higher level of the expression of acetylation leads to the reversal of the deacetylation of histone protein and protects from the DNA methylation [25]. The methylation of histone mainly happens at the lysine or arginine residue with addition of either one to three methyl groups. The methylation of histone protein is related with both activation and repression of the transcription which depends upon the region of the gene, level of the modification. In transcription activation, the lysine 4 residue of histone 3 is either in monometylated or trimethylated form (H3K4me1 and H3K4me3) and trimethylation of the lysine residue 9 and 27 of the histone 3 (H3K9me3 and H3K27me3) are related with silencing and inactivation of gene. The methylation of DNA mainly happened with absence of H3K4 and presence of the H3K9 [26]. The important connection between histone methylation and DNA methylation was DNMT protein; it mainly binds to the H3, when it is in unmethylated form. The histone methylation of H3K4 leads to begin *de novo* methylation of DNA, which results in the transcription activation. The interaction of the DNMT to the H3K4 on the DNA at promoter level leads to transcription repression. The interaction of H3K9 with DNA methylation recruited with ubiquitin-like, containing PHD and RING finger domains, 1 (UHRF) protein which increases the attachment of the DNMTs to the DNA, which results in transcription repression [27] (Figure 1).

Histone modification of the protein which is involved in the DNA packaging into the chromatin, leads to instability in chromatin structure and abnormal DNA repairs. The acetylation level of lysine 9 of H3 histone protein (H3K9Ac) in the type 1 diabetes susceptible genes, HLA class II histocompatibility antigen, DRB1 β chain (HLA-DRB1) and HLA-DQB1, was found to be higher in T1D patients as compared with the normal individuals [28]. The methylation level of H3K9me2 of CLT4 in lymphocytes was found to be significantly higher in comparison with the control person [29].

### miRNA dysregulation

miRNAs control the gene expression by influencing the stability and translation of messenger RNAs (mRNAs) via straight inhibition and degradation of mRNA. Epigenetic changes of miRNAs have been related to the cell cycle modification with alteration in immune reaction. The reports of miRNAs expression in the Treg cells of type1 diabetes patients showed over-expression of miRNA-146a and under-expression of miRNA 20b, 31, 99a, 100, 125b, 151 and 365 which resulted in direct involvement of miRNA in the immune response regulation that leads to T1D [30]. In animal studies, the over-expression of miRNA 21, 34a, 146a and 29 leads to the β cell destruction by release of pro-inflammatory cytokines. The over-expression of miRNAs results in the insulin resistance, decreased expression of Onecut2 transcription factor [31,32] and increased level of granuphilin confirmed that miRNA expression level is directly associated with β-cell functioning in humans. Studies in human were conducted comparing age matched healthy controls with miRNA serum level from new onset T1D children, miRNAs expression levels were found to be associated with glycemic control and β-cell function. Twelve human miRNAs were found to be up-regulated in T1D patients, *viz* miR – 152, 30a-5p, 81a, 24, 148a, 210, 27a, 29a, 26a, 27b, 25, 200a. Moreover, miRNA-25 was positively correlated with glycemic control (hemoglobin A1c or glycated hemoglobin, HbA1c) (*P*=0.0035) and negatively correlated with residual β-cell function (*P*=0.0037), several miRNAs were also linked to apoptosis and β-cell function [33].

In spite of this, the monogenic configuration of diabetes remains unidentified. Molecular diagnosis has raised awareness about the genes that control β-cell function and their total analysis will further identify the fundamental pathways involved in the pathogenesis of diabetes [34,35]. The uncommon conformation of monogenic diabetes is equally important as clinically and scientifically because its diagnosis helps in the prevention and management of T1D. For this reason, it is necessary to accurately diagnose monogenic diabetes [36]. T1D mainly accounted by an interaction between environment and genetic factors, a major factor involved is Class II human leukocyte antigen (HLA) in T1D which is responsible for the genetic changes in children and young adults. HLA class II involves HLA-DRB1, HLA class II histocompatibility antigen, DQ α 1 (HLA-DQA1) and HLA-DQB1 genes [37], present at HLA locus on chromosome 6p21, which encodes for antigen-presenting proteins. DR3-DQ2 and DR4-DQ8 are majorly present in 90% of the total population of children in T1D [38]. In histocompatibility complex class II the DQA1*0102-DQB1*0602-DRB1*1501 alleles which are directly connected with T1D protection [39,40]. The immunity provided by DQA1*0102-DQB1*0602 is superior to the disorder caused by all other DQ haplotypes. This protection helps the children even in the presence of B-cell antibodies [41].

In the case of early-onset diabetes (EOD) is described by significant dissimilarities between the frequency of haplotypes and HLA alleles. Hathout et al. [42], studied the above mechanism on 40 patients (age less than 2.6 years), none of the protective alleles in patients were found in his study. In EOD study, a negative association was found between glutamate acid decarboxylase 65-kilodalton (GAD65) and DR3/DQ2 haplotypes. Other accepted genetic loci that connected with T1D risk have a minimal effect than HLA, confirmed by relative risk vary from odds ratio (OR) = 2.38 (11p155.5, insulin gene (INS)) to OR = 1.05 (17q21.2: SWI/SNF-related matrix-associated actin-dependent regulator of chromatin subfamily E member 1 (SMARCE1)) [43–51]. Apart from this study, new array-based genotyping technique, genome-wide association studies (GWASs) discovered several new replicated loci [43–48]. GWASs technique established 140 new polymeric variant region which has a strong connection with acquired immune deficiency syndrome (AIDS) and T1D [51,52]. It was identified that ∼44% of single nucleotide polymorphisms (SNPs) connected with T1D and AIDS has an association with other complications. This is an example of biological pleiotropy [53]. The ordinary failure in the function of protein tyrosine phosphatase, non-receptor type 22 (PTPN22), it minimizes the risk of Crohn’s disease but maximizes the risk of T1D and AIDS [54]. Several relative studies were performed with different types of SNPs to estimate the genetic variation in profiles of ankylosing spondylitis, an autoimmune disorder, Crohn’s disease, multiple sclerosis (MS) and T1D with five different non-AIDS. The present ustudy helps to identify the group of alleles in two different types of disease with opposite risk [52]. The class II HLA has DR and DQ locus present in T1D also shared by other diseases like rheumatoid arthritis (RA), systemic lupus erythematosus, MS and celiac disease. Study on T1D risk locus and celiac disease has been done on a large number of patients, evidence was found that the T-cell activation RhoGTPase activating protein (TAGAP) locus, which are strongly related with celiac disease but has a protective effect on T1D [55,56]. Meta-analysis study was found that SNPs have multiple types of relationships in different diseases. The study has been expanded to protein–protein interactions and verified that the protein variant encoded for one group were strongly related to each other than compared with other groups; this provides the efficient proof of biological pleiotropy in AIDS [57]. The diagnostic chip for AIDS, which has several common loci contain T1D, to analyze the rare and common variant in different disease and mapping of important genetic locus the array was established which has 20000 known SNPs are present and to find the novel SNPs it is necessary to re-evaluate the data [20]. To find out the pathogenesis of T1D by applying GWAS data, it is necessary to put the integrative pathway-based approach. Apart from it, next-generation sequence data from 1000 Genome projects helps to discover the four important variants in the interferon induced with helicase C domain 1 (IFIH1) gene [48,50]. By combining two above approaches (GWAS study and next-generation sequence) gives the hypothesis about the involvement of genetic–virus inter-relation and increased level of Type 1 interferon as a cofactor in β-cell destruction [43]. A recent study has found out that the increase in the incidence of T1D contain lifestyle, dietary factors, socioeconomic factors, environment pollutant and prenatal factors.

**Figure 2:**
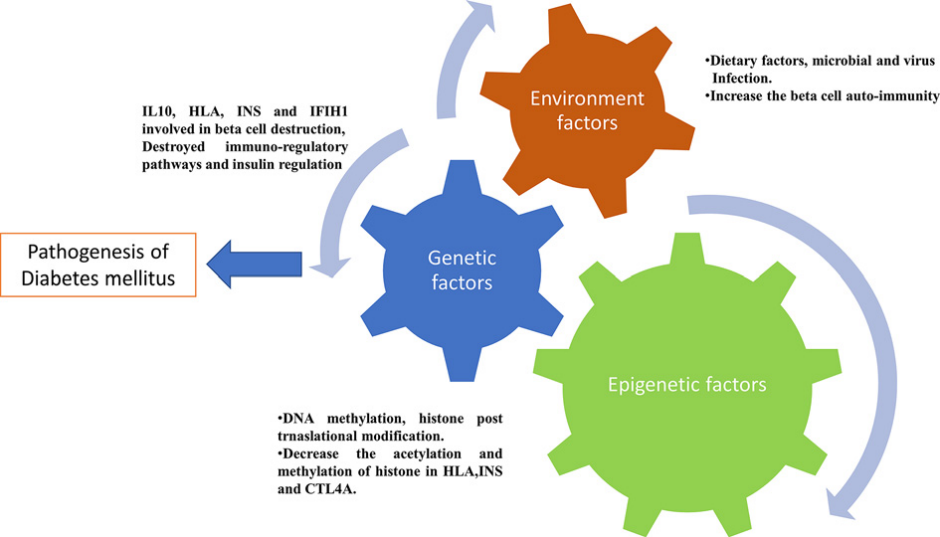
Factors involved in Type 1 diabetes pathophysiology

From these findings, it is cleared that compilation of epigenetic changes with genetic and environments factors plays a critical role in pathogenesis of T1D (Figure 2). Moreover, the epigenetic factor interplay the role between genetic and environmental factor to triggers the onset of T1D. These changes are reversible in nature, so it can be used for management of T1D.

## Epigenetic modification responsible for diabetes mellitus type 2

The frequency of T2D has increased significantly from several decades, estimated to extend to 366 million around the world by 2030 [58]. Mendelian inheritance manner unable to discover the basis of T2D genetics, in the present study they tried to identify the mutation with the help of SNPs but failed to identify it. Currently, the GWAS study discovered 17 loci linked with T2D [59], which describe the T2D affected by the interplay between several genetic environmental mental factors. The key environmental factors involved in T2D contain Intra-Utero environments, obesity, low birth weight, and advancing age [60]. These environmental factors affect gene expression that leads to T2D by epigenetic modification. The major factor that involved in T2D was intrauterine growth retardation (IUGR). IUGR mainly affects the gene expression of pluripotent cells that replicate rapidly and also affects differentiated cell that replicates poorly. The effects of IUGR on adulthood depend upon the time and state of exposure to cells during its proliferation, differentiation and maturation. There are a lot of examples in which IUGR directly associated with T2D such as—during World War II, a pregnant woman’s intake only 400–800 calories per day gave birth to a child with low birth weight. When a child becomes 50 years old, it was diagnosed with impaired glucose tolerance compared with other children that are *in utero* either before or after the famine [61]. Another study from Hertfordshire, U.K. established that persons that were low birth weight (less than 2.5 kg) were seven-times more prone toward the development of T2D [62]. IUGR related to a stable and permanent change in gene expression of the child which leads to epigenetic modification. In this part, we provide information about the possible mechanism of epigenetic modification in the development of T2D.

Epigenetic modification of genome leads to permanent proliferation of gene expression from one generation to another generation [63–71]. Mainly two different mechanisms are involved in an epigenetic modification in Type2 diabetes such as—histone modification and DNA methylation.

### Histone modification

**Figure 3:**
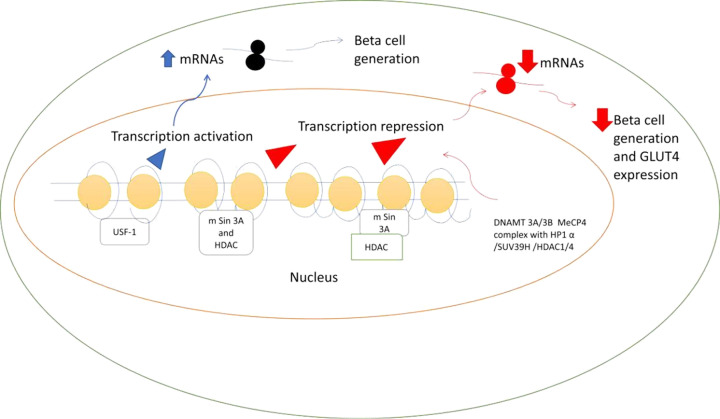
Epigenetic modification in IUGR islet In pancreases, Pdx1 region remains in the unmethylated form, the nucleosome is in the acetylated form at H3K4, which leads to recruitment of USF-1 transcription factor, resulted in Pdx1 expression and generation of β cell. In IUGR fetal and IUGR 2-week islets, the association of mSin3a-HDAC-DNAMT1 to the histone leads to dimethylation of H3K9, which leads to Pdx1 repression and this complex also leads to inactivation of chromatin with dimethylated of H3K9 which resulted in pdx1 transcription silencing and decease in β-cell generation. In IUGR adult muscle, histone acetylation was lost, due to the recruitment of HDAC1 and HDAC4, but methylation occurs on CpG island. But CpG island directly related with complex of repressor DNAMT3A, DNAMT3B and MeCP2. This makes the recruitment of suv39H1 which leads to methylation of H3K9 and its enhanced the recruitment of HP1α, that inactive the GLUT4 expression. Abbreviations: DNAMT3A/B, DNA methyltransferase 3 α/β; GLUT4, glucose transporter type 4; suv39H1, suppressor of variegation 3-9 homolog 1.

Histone modification in eukaryotes cells contains four histone proteins, i.e. H2A, H2B, H3 and H4. The lysine residue of this protein can be easily modified by several mechanisms such as acetylation, methylation, phosphorylation, glycosylation and adenosine diphosphate (ADP) ribosylation. The main mechanism involved in the epigenetic modification is acetylation and methylation at lysine residue on amino-terminal. In the epigenetic modification the main mechanism involved is acetylation and methylation at the lysine residue that is present on amino-terminal. The increased acetylation of a lysine residue on amino-terminal induces increased transcription, whereas, decreased acetylation induces repression of transcription [63]. In methylation mechanism, it is related to both increased and decreased in transcription level. *In vivo* lysine group present at amino-terminal may be mono-di and tri-methylated. This gives an extra level of regulation [63]. Recently, one study has been carried out on IUGR rat which described the pancreatic and duodenum homeobox 1 (Pdx1) expression. Pdx1 is a homeodomain-containing transcription factor mainly involved in the early generation of both exocrine and endocrine pancreases, later involved in the development of β cell. In early detection in 24 h in IUGR rat was found a 50% decrease in Pdx1-mRNA expression. Repression of Pdx1 was found after birth and significantly decreased with time in IUGR rat, which indicates the epigenetic changes. The first indication of histone acetylation was found in β cell in IUGR rat, β cell has been isolated from pancreas indicates it reduces the level of H3 and H4 at Pdx1 promoter [72]. These epigenetic changes at H3 and H4 acetylation were related with the binding of upstream transcription factor 1 (USF1) to PDX1 promoter region. USF1 is required for activation of pdx1 transcription, a decrease in binding of USF1 to pdx1 leads to pdx1 transcription silencing [73,74]. Increase in histone deacetylation leads to decrease in trimethylation of H3K4 and increase in H3K9 dimethylation in IUGR rat [72] (Figure 3). This resulted in chromatin gene repression. These factors collectively involved in unbalancing of glucose homeostasis and induce oxidative stress condition in IUGR.

Reversal of histone de-acetylation is possible in 2 weeks of age in IUGR rat which leads to normalizing the Pdx1 mRNA level which helps in generation of β cell [72]. In IUGR rat, the recruitment of HDAC1 and mSin3A to histone protein acts as corepressors, this resulted in Pdx1 silencing. This complex increases the loss of trimethylation of H3K4, which facilitate the Pdx1 repression. This complex can be dissociated by applying HDAC inhibitor (Trichostatin) reversal the Pdx1 silencing [72].

### DNA methylation

The important mechanism involved in DNA methylation in islet is mainly dependent on the level of methylation of lysine 9 on H3 (H3K9). In studies it was found that methylation at lysine 9 anticipated in changes of DNA methylation [75,76]. It has also been proposed that DNA methyltransferase can proceed on histone protein where H3K9 is methylated [77]. Additional enzymes such as DNA methyltransferase 3A and 3B, attached to methylase to start DNA methylation [78]. This process starts the epigenetic modification in IUGR islet, in which HDAC/mSin3A complex binds to the Pdx1 that leads to deacetylation and repression of Pdx1 transcription. The repression and disruption of Pdx1 lead to pancreatic agenesis and involved in some type of mutation in human phenotype [79]. Milder depletion in Pdx1 protein level, enable the usual β cell mass, besides it leads to impairment of several factors involved in insulin secretion from β cell. This epigenetic modification pathway provides the therapeutic window for the discovery of novel agents for the prevention of T2D. Histone modification also found in the muscle of IUGR rat which is the indication of insulin resistance [80,81]. Under normal conditions, glucose is transported from blood to cell by passive diffusion [82]. The transporters involved in the transportation of glucose from the blood into the cell is GLUTs; slc2 family, glucose transporter type 4 (GLUT4) is the major form of GLUT family mainly found in skeletal muscles, adipose tissue and cardiac muscle [83]. myoblast determination protein (MyoD) and myocyte enhancing factor 2 (MEF2) factors involved in an increment of GLUT4 expression, it binds to 502 to 420 bp region with TR1 at GLUT4 in skeletal muscle [84].

A study by Raychaudhary et al. [85], found that increased expression of MEF2D (the second form of MEF family acts as inhibitor) and decrease in expression of both MYOD and MEF2A which resulted in repression of GLUT4 expression. Another pathway involved in IUGR Glut4 transcription was contained H3K14 (histone 3 lysine 14) deacetylation, which is processed by attachment of HDAC1 and increased recruitment of HDAC4. This complex helps to the recruitment of suppressor of variegation 3-9 homolog 1 (Suv39H1) methylase which leads to dimethylation of H3K9 and increased attachment of heterochromatin protein 1. This resulted in IUGR GLUT4 gene repression (Figure 3). These types of studies explain about the perinatal nutrition deficiency leads to IUGR produces histone modification which ultimately results in decreased GLUT4 expression and glucose transportation [85]. Brasacchio et al. demonstrated the mechanism of how hyperglycemia increased the histone modification at the region of NFkB-p65 in vascular epithelial cells, effects p65 expression and leads to vascular complications [86].

## Epigenetic therapeutic targets

### Epigenetic molecular drug targets

Epigenetic modification mainly contains Histone and DNA modifications, which leads to severe phenotype changes—this part is mostly entertained because of these events, are inherently reversible and this is advantageous for selecting the epigenetic targets. In respect to environmental and genetic stress, epigenetic changes may proceed ahead or backward, after withdrawal of stress it reverts back to original state. Recent studies has shown that histone modification mainly contribute toward methylation of CpG island, which relate the several epigenetic changes and regulations [87]. This relation makes a new framework where an epigenetic code dominates the level of particular genes which fundamentally employed as on/off switch for several cellular components [88]. The continued efforts have to be placed for developed the novel drug candidate to control specifically to these types of switches. When correct combination of drug candidate has been selected for this switches then we can easily employ these switches to reversal back the phenotypes changes specially when these drugs have been administered at early progression of disorder.

Focus on new and novel management for diabetes mellitus, the probability of utilizing the reversibility of epigenetic modifications for regulation of glucose metabolism has been fascinated. In facts, the several novel small molecules have been established with epigenetic activity [89]. These molecules have been classified according to their epigenetic effects which called as epidrugs. Several molecules with epigenetic activity are well known and some are under clinical trials. In case of chromatin modification, these are histone acetyltransferase inhibitors, HDAC inhibitors and RNA interference molecules.

### HDACs

**Figure 4:**
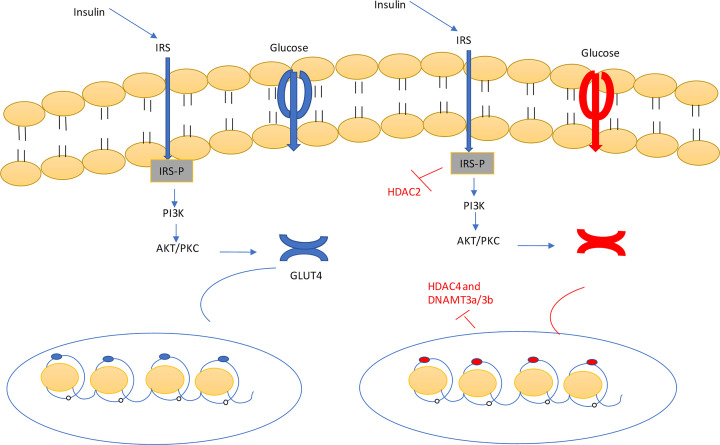
Insulin binds with the insulin receptor, leads to phosphorylation of the receptor, which activates the PI3K/AKT pathways Same time, the acetylation of histone protein, leads to attachment of myoD and Mef2a which emphasizes the release of the GLUT4. GLUT4 transfers the glucose from the outside cell to inside of the cell. Under the influence of HDACs (HDAC2 and 4) and DNAMT3A/3B, the histone deacetylation and hypermethylation of histone protein occurred. Which resulted in decrease or inhibition the expression of GLUT4. HDAC and DNAMT3A/3B inhibitor blocked these enzymes and helped in reversal the mechanism into normal conditions. Abbreviation: DNAMT3A/3B, DNA methyltransferase 3 α/β.

Now it is well known that T2D mellitus does not contain only genetic factors that related to etiology of disorder [90]. Beside from this, epigenetic factors may play important role in etiology of diabetes mellitus. In epigenetic mechanisms involved in the etiology of diabetes, histone acetylation and histone deacetylation have been broadly described. The studies have been described that HDAC and HAT regulation are related to signaling of insulin [91], apoptosis and inflammation [92–94] which has effect on the both type of diabetes. The major case of HAT and HDAC effect on the diabetes was laid on the molecular mechanism of insulin transcription mediated by Pdx1. Pdx1 is the key regulator involved in insulin transcription [95]. When body has hyperglycemia, it leads to increase the association of Pdx1 with p300 HAT, which involved in the enhancement the histone acetylation and insulin transcription. In opposite condition, in hypoglycemic condition the pdx1 enhanced the association of HDAC, which leads to change in chromatin structure and decrease in insulin transcription [96,95]. When pdx1 faces specific mutation, it leads to impaired β-cell functioning which resulted in T2D mellitus [95]. Hence, the participation of HDACs in the etiology of diabetes makes this as new therapeutic targets for the management of both types of diabetes mellitus [91,96]. Then HDAC inhibitors was invented for the regulation of insulin signaling and β-cell functioning. Examples of HDACi are trichostatin A, suberoylanilide hydroxy acid, valproic acid, sodium butyrate and givinostat [91,96] (Figure 4).

### DNA methyltransferase

Diabetes mainly developed due to lack of insulin release from the β cells and the abnormally high production of glucagon from the α cells of pancreas. Several studies have been found that DNA. Methylation plays the vital role in functioning of pancreatic islet cells [97]. The important study was conducted to analyze the important factors and gene that was involved in functioning of β cell. These genes included the INS, Pdx1, peroxisome proliferator-activated receptor γ co-activator 1-α (PPARGC1A) and GLP1R which were hypermethylated in case of diabetic patients [98–102]. Moreover, hypermethylation leads to reduce the expression of specific gene in diabetic islet and with enhanced level of HbA1C which is involved in β-cell disruption in T2D. Hyperglycemia leads to increased DNA methylation at Pdx1 and INS in β cell [98,99].

### Sirtuin 1

**Figure 5:**
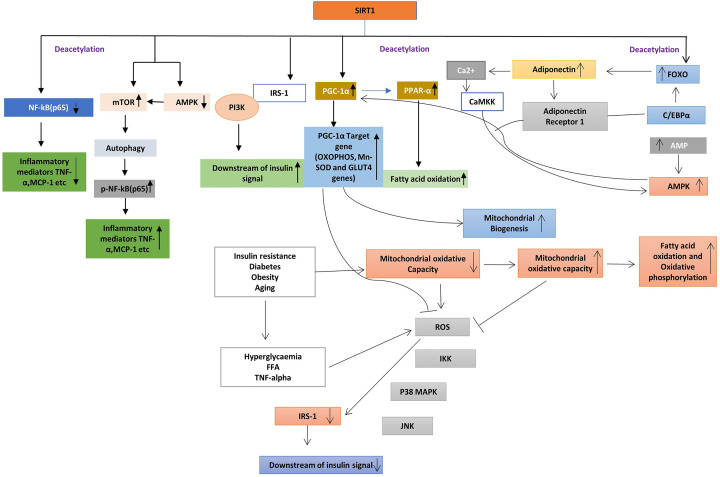
In adipocytes/macrophages, SIRT1 deacetylation leads to a decrease in the expression of several inflammatory mediators such as MCP-1 and TNF-α SIRT1 inactivation leads to NF-kB phosphorylation related to activation of mammalian target of rapamycin (mTOR) and decrease in expression of AMP-activated kinase (AMPK). In adipocytes, SIRT1 deacetylates the nuclear factor NF-kB p65, leads to a decrease in the expression of TNF-α and MCP-1. In skeletal muscle, SIRT1 induces the expression of peroxisome proliferator-activated receptor (PPAR-) co-activator 1α. Under diabetes condition mitochondrial oxidative capacity reduced which leads to the generation of free radical oxygen species, free fatty acids and TNFα reduced the insulin signaling via insulin receptor substrate phosphorylation. SIRT1 also activate phosphoinositide 3-kinase (PI3K). SIRT1 activates the PGC-1α induces the mitochondrial biogenesis which reduced the generation of ROS, increase the generation of GLUT4. SIRT1 deacetylates the forkhead box protein O1 (FOXO1) and increased the recruitment with CAAT/enhancer-binding protein (C/EBPα) which increase the generation of adiponectin1 in adipocytes. Adiponectin also activates the calcium/calmodulin-dependent protein kinase kinase (CaMKK) and calcium/calmodulin-dependent protein kinase kinase (CaMK). Adiponectin activates the SIRT1 via AMPK pathway activation. Its leads to deacetylation of PGC-1α resulted in mitochondrial biogenesis.

It is a member of the sirtuin family, also called as NAD-dependent deacetylase and evaluate in bacteria [103]. The seven types of sirtuin are present in human beings mainly present in nucleus and cytoplasm of a human cell. It has several roles in metabolic disorder, its deacetylates the uncoupling proteins and PPARG co-activator-1 α (peroxisome proliferator activated receptor γ co-activator 1 α). The uncoupling protein 2 (UCP2) is main uncoupling protein present on adipose tissue involved in negative regulation of insulin secretion and PPARG co-activator-1 α is a main key factor in glucose production in the liver and involves in regulating the gluconeogenesis pathways [104]. Evidence has described that Sirtuin 1 (SIRT1) decrease the inflammatory process by affecting the NF-kB signaling pathways via deacetylating the p65 subunit of Lys^310^ [105]. In macrophages and adipocytes, this deacetylation resulted in enhancement of glucose metabolism [106,107]. Furthermore, SIRT1 knockout mice fed with a high-fat diet leads to activation of macrophages and another inflammatory mediator in the liver which related to the development of insulin resistance [108]. SIRT1 in adipose tissue regulates the expression level and secretion of adiponectin, tumor necrosis factor α (TNF-α), monocyte chemoattractant protein 1 (MCP-1) and interleukin 4 (IL-4) which altered the attachment of macrophages at adipose tissue. SIRT1 increases the expression of IL-4 via deacetylating the transcriptional factor, nuclear factor of activated T cell, cytoplasmic 1 (NFATc1), resulted in the polarization of M2 subtype of macrophages [109]. Hence, SIRT may decrease the inflammation in macrophages and adipose tissue and it may increase the insulin sensitivity. In a study, the expression of SIRT1 is reduced in myotubes and skeletal muscle isolated from T2D patients, who suggested the down-regulation of SIRT1 related to insulin resistance evoked by TNF-α in skeletal muscle [110]. SIRT1 assembled to p85 subunits of PI3K which triggers the insulin signaling in skeletal muscle [110]. Also, SIRT1 helps to preserve the β cell from various oxidative stresses, reactive oxygen species and by suppressing NF-pathways. SIRT1may participate in the regulation of insulin resistance via modulation of the functioning of mitochondria. The PPAR-α conserved the biogenesis of mitochondria and oxidative phosphorylation (OXPHOS) protein, which involved in competent β oxidation of fatty acid in skeletal muscle. In T2D the expression of peroxisome proliferator-activated receptor 1-α (PGC-1α) is down-regulated. SIRT1 regulates the metabolic and mitochondrial homeostasis, which increment the oxygen consumption in skeletal muscle and increased the expression of OXPHOS genes. The biogenesis of mitochondria is maintained by deacetylation of PGC-1α, knockdown of SIRT1 involved in down-regulation of PPAR1α which is involved in fatty acid utilization [111]. This deacetylation of PPAR1α resulted in increased fatty acid oxidation (Figure 5).

Therefore, SIRT1 can increase the insulin resistance via promoting oxidation of free fatty acid and biogenesis of mitochondria through deacetylation of PPAR-α and PGC-1α activation of skeletal muscles. Therefore, the PGC-1α significantly increased the expression of GLUT4 which resulted in transport the glucose C2C12 murine myotubes [112]. Adiponectin is a protein hormone, which has the anti-diabetic property [113], the expression of this hormone is decreased in insulin resistance and T2D [114,115]. Management with adiponectin leads to a decrease in glucose level and recovered the insulin resistance in mice. Additionally, adiponectin increased the insulin sensitivity by enhancement of fatty acid oxidation by AMP-activated kinase (AMPK) and PPARα pathway activation [113]. SIRT1 deacetylation leads to increase the expression of forkhead box transcription factor class O1 (FOXO1), which emphasizing the recruitment with CCAAT/enhancer-binding protein a (C/EBPa), that resulted in an increase the transcription level of adiponectin gene in adipose tissue [116]. The adiponectin plays an important role in skeletal muscle by regulation of Ca^2+^ signaling pathway and also activate the PGC-1α in adiponectin receptor 1 knockout mice [117]. Adiponectin binds to adipoR1 receptor leads to activation of AMPK, resulted in further activation of SIRT1, deacetylation of PGC-1α which enhances the mitochondrial functioning, lipid/glucose metabolism [117], which resulted in enhancing insulin sensitivity. SIRT1 induced deacetylation of PGC-1α resulted in mitochondrial improvement by mitochondrial biogenesis, adiponectin and GLUT4 induction which collectively increased the insulin sensitivity and prevent T2D.

### Protein tyrosine phosphotase 1B

Protein tyrosine phosphotase 1B (PTP-1B) comes under the family of PTP enzyme family having 435 amino acids, encoded by PTpn1 genes with a molecular weight of 50 kDa [118,119]. Protein-tyrosine phosphatase 1B (PTP1B) with unphosphorylated form having several functions such as—cell differentiation, growth and apoptosis [119]. PTP1B has mainly two domains, N-terminal domain and C-terminal domain. The N-terminal domain contains two aryl phosphate-binding sites called a high-affinity catalytic site which consists of cysteine residue and non-catalytic non affinity site having Arg^254^ and Arg^24^ [120], although, C-terminal contains proline and hydrophobic amino residues. In the insulin pathway, PTP-1b dephosphorylated the tyrosine residues 1162 and 1163 which resulted in discontinuation of tyrosine kinase receptor cascade. The attachment of insulin with insulin receptor leads to IR phosphorylation which resulted in the activation of secondary messengers which helps in the regulation of glucose homeostasis [121]. PTB1B commands the interplay between IRS1 and IRS2 that regulates the action of insulin in the liver and several tissues [122]. Hence, PTB1B has an important role in insulin resistance, as it is a crucial drug target in the management of T2D.

## Other molecular therapeutic targets

The management of diabetes and its complications is not an easy task. The management of diabetes can be resolved by identifying the novel therapeutic targets and discovery of new drugs several hormones are involved in glucose homeostasis in the human body such as incretin, glucagon-like peptide-1 (GLP-1) and glucose-dependent insulin trophic peptide (GIP). The incretin is degraded by dipeptidyl peptidase-IV enzyme, inhibition of this enzyme leads to lowering of the blood glucose level [123]. Over-expression of glycogen synthetase kinase 3 is responsible for insulin resistance in human beings. Selective glycogen synthase kinase 3 (GSK3) inhibitor leads to increase in the insulin sensitivity in skeletal muscles [124]. Discovery of new targets and understanding of proper pathway are required to the establishment of new drug candidates in the market for the management of diabetes.

### Dipeptidyl peptidase 4 inhibitors

**Figure 6:**
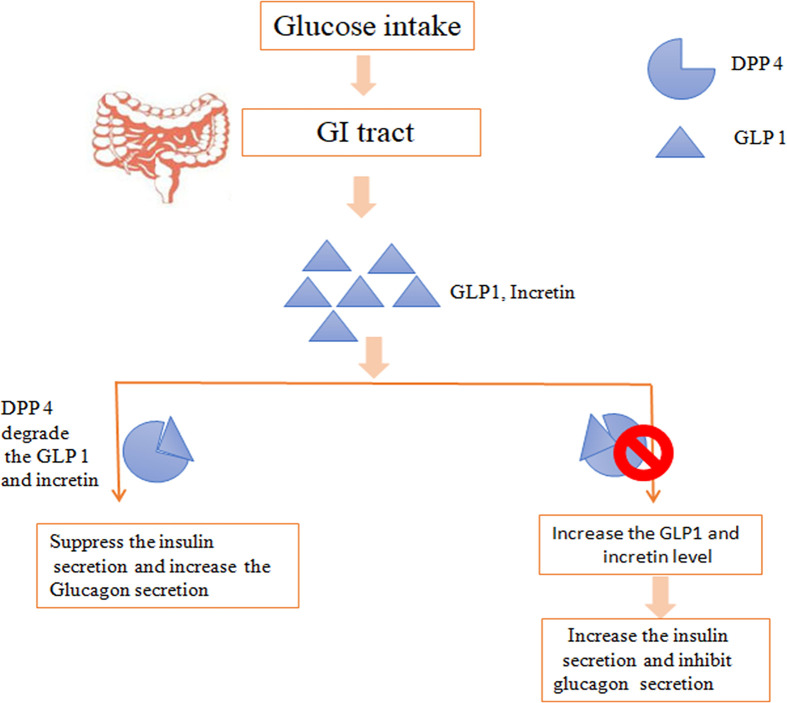
DPP4 degrades the GLP1 and incretion, which leads to suppression of the insulin secretion DPP-4 inhibitor inhibits the DDP4 enzyme, which resulted in increase the level of GLP1 level and increases the insulin secretion.

The primary hormones involved in metabolism is GLP1, after taking a meal, GLP1 is released with GIPs which increased the release of insulin from β cells and inhibits the glucagon secretion [125]. In susceptibility toward diabetes, dipeptidyl peptidase-4 (DPP-4) degrades the incretin and involve in insulin resistance [126]. DPP-4 is soluble, type II transmembrane glycoprotein present on most of the cell and related to signal transduction, immune regulation and apoptosis. Hence, management therapy has been implemented as DPP-4 inhibitors for inhibiting the degradation of incretin (Figure 6).

### Peroxisome-proliferator activated receptor-γ

PPAR-γ is a nuclear receptor (Type 2) mainly found in adipose tissue [127], identified as an anti-diabetic target by Choi et al. in a year [128]. PPAR-γ expressed mainly in three isoforms at separate locations. PPAR-γ2 and PPAR-γ3 significantly found in large number in adipose tissue and skeletal muscle but PPAR-γ1 is less in level. Expression of PPARγ was found to increase with insulin resistance [129,130]. The expression of PPAR-γ is maintained by glucocorticoids and tumor necrosis factors [131]. The activation of PPAR-γ leads to increase in the uptake of glucose from the blood to adipose tissue. The drugs/agonists such as thiazolidinedione attached to PPAR-γ receptor which resulted in activation of receptor and formed the complex with important transcription factor retinoid-X receptor (RXR). This complex then attaches with particular DNA motif at promoter region in target gene [132], which resulted in glucose homeostasis by increasing the insulin sensitivity and GLUT4 expression. The direct stimulation of PPARγ resulted in inactivation of lipoprotein lipase and fatty acid transporter 1 which leads to a decrease in the FFA level and triglycerides [133]. Tumor necrosis factor and free fatty acids are involved in the development of insulin resistance, and then the activation of PPARγ contributed toward down-regulation of both parameter and increased the insulin sensitivity [134]. Several attempts have been done for identification of genes involved in insulin.

**Figure 7:**
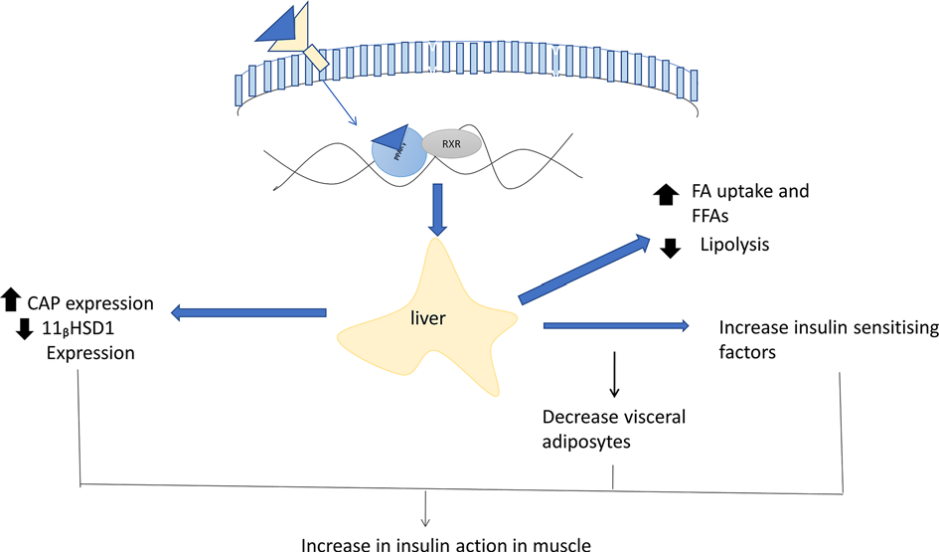
Insulin sensitivity mechanism of PPARγ ligands, PPARγ mainly expressed in adipose tissue Ligands binding to the receptor led to a specific change in the expression of a gene. Inhibition in the expression of an adipose-related gene such as, fatty acid transporter that inhibited the production of free fatty acids which resulted in an increase in the insulin sensitivity. The change in expression of genes such as CAP or 11βhSD1 helped to increase the insulin action in adipose tissue and decrease adipose visceral fat. Other factors TNF-α, resistin, and Acrp30 were also involved indirectly in insulin sensitivity in adipose tissue. Abbreviations: 11β-hydroxysteroid dehydrogenase type 1; Acrp30, adipocyte compliment protein 30; CAP, catabolite activator protein.

The identified a novel protein resistin which is involved in insulin resistance. Rosiglitazone was the first thiazolidinedione drug which decreased the expression of resistin but the proper mechanism was unknown [135]. Pyruvate dehydrogenase kinase 4 (PDK4) gene expressions were successfully suppressed by PPAR^γ^ agonists *in vivo* in the rat [136]. The final results for inhibiting the PDK4 increases the level of pyruvate dehydrogenase which results in glucose utilization. In peripherally, 11-b hydroxysteroid dehydrogenase type 1 (11bHSD1) is the enzyme that involved in the conversion of cortisone into active cortisol. Hence, the inhibition of this enzyme is also a potential drug target in metabolic syndrome [137] (Figure 7).

### Sodium-glucose linked transporter

Diabetes is a metabolic disorder with multifactorial etio-patho-physiology. Insulin resistance is the main reason behind the development of diabetes and resulted in a decrease in the secretion of insulin from β cell of pancreas. The insulin resistance leads to an increase in the secretion of glucagon, breakdown of lipids and increased the re-absorption of glucose from nephrons. Glucose is the main source of energy in human beings; glucose is mainly transported by two transporters known as GLUT and sodium-glucose linked transporter (SGLT). The sodium-glucose transport transfers the glucose from lumen to blood against the concentration gradient. Mainly sodium-glucose linked transporter 1 (SGLT1) and 2 (SGLT2) is the main co-transporter in the lumen of the kidney which facilitates the re-absorption of glucose. Inhibition of these two co-transporters leads to decreases in the re-absorption of glucose to blood and promote glycosuria. In normal human beings, 95% filtered glucose is reabsorbed by these transporters, which helps to maintain glucose homeostasis [138].

**Figure 8:**
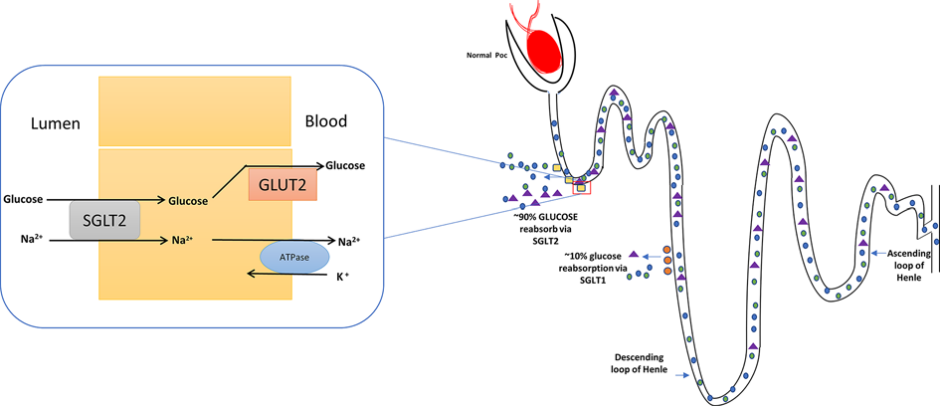
Glucose re-absorption by glucose transporter in a normal individual and diabetic person SGLT2 is major co-transporter for the glucose re-absorption present at apically in epithelial cells of PCT. It reabsorbs ∼90% of glucose from outside to the blood and ∼10% glucose is reabsorbed by SGLT1. For re-absorption of glucose, these two co-transporters need ATP for active transportation of sodium-potassium. This exchange resulted in re-absorption of glucose via GLUT. The inhibition of these co-transporter leads to inhibit the re-absorption of glucose into blood which resulted in excretion of glucose by urine.

In T2D patients, these transporters are up-regulated which increase the transportation of glucose into the blood. Currently, SGLT2 inhibitors have their anti-hyperglycemic effect on various animal models [139]. SGLT1 reabsorbs approximately 10% of glucose from the lumen into blood [140] (Figure 8). The problem with inhibition of SGLT1 resulted in glucose malabsorption, which leads to gastrointestinal symptoms [141]. The selective SGLT2 inhibitor has been designed to decrease the above symptoms and renal re-absorption of glucose. The selective SGLT2 inhibitors lead to reduce the renal glucose re-absorption and increase the excretion of glucose through urine, this phenomenon helps to increase insulin sensitivity and β-cell functioning [142,143].

## Role of long non-coding RNAs and cirRNA in diabetic complications

### Long non-coding RNAs

Long non-coding RNAs (lncRNAs) are a group of RNAs ranging from 200 nts to 100 kb in length and possess a potentiality of limited protein-coding and functionality as regulatory genes at the epigenetic, transcriptional and post-transcriptional levels. In last several years, scientific evidences are totting up to show that lncRNAs can regulate a variety of important biological processes and are also involved in development of a variety of diseases, including metabolic diseases like diabetes and other diseases like depression. In diabetes, some recent works has provided the evidence that lncRNAs are often dysregulated during pancreatic cell differentiation, as well as by hyperglycemia and other related growth factors. They can also play role in diabetic complications by incorporating changes in inflammation, fibrosis, ER stress, mitochondrial dysfunction and oxidant stress. Few studies suggested the involvement of lncRNAs in diabetes and diabetes related complications by showing that a number of human SNPs associated with type 1 diabetes and T2D are located within the lncRNA loci [173]. Arnes et al. showed that a conserved lncRNA named βlinc1 (β-cell long intergenic non-coding RNA 1) is involved in coordinated regulation of a variety of islet-specific transcription factors located in the genomic vicinity of βlinc1 that are essential for the specification and function of insulin-producing β cells. Furthermore, it was also observed that deletion of βlinc1 results in defective development of islet and disruption of glucose homeostasis in adult mice [174]. Yin et al. has reported that in pancreatic tissue another lncRNA TUG1 was highly expressed compared with other organ tissues, and in Nit-1 cells its expression was vigorously regulated by glucose. TUG1 was observed to be an important regulatory factor for the function of pancreatic β cells as its down-regulation of expression affected apoptosis and insulin secretion in pancreatic β cells *in vitro* and *in vivo*. lncRNA-p3134 is associated with glucose metabolism and insulin signaling in pancreatic β cells and its circulating level was high in diabetic patients [175]. Its over-expression in Min6 cells showed an increase in glucose-stimulated insulin section that is consistent with the up-regulation of insulin transcription factors. Studies have revealed that the transcription factors Pdx-1 and MafA stimulate the insulin gene promoter in response to elevated blood glucose which finally leads to stimulation of insulin synthesis [176]. In high glucose conditions a lncRNAMalat1 was found to be up-regulated in retinas while Malat1 lncRNA knockdown in STZ induced rats resulted impaired retinopathy [177,186] suggesting that this lncRNA promotes retinopathy under diabetic condition. Furthermore, increased expression of linc-MIAT lncRNA was observed in retinal endothelial cells in presence of high glucose. This lncRNA can regulate VEGF levels or suppress the miR-29b, functioning as a competitive endogenous RNA of miR-150 in regulating apoptosis [178–180]. Molecular mechanisms of action of many lncRNAs, their protein binding partners and genomic targets needs to be identified further to study the disease biology more clearly. Expression analysis of lncRNAs in biofluids can be a valuable non-invasive biomarker for early detection of diabetic complications which is now a major unmet need in clinical management. lncRNA growth arrest-specific 5 (GAS5) and lncRNA ENST00000550337 can serve as a potential diagnostic biomarker for pre-diabetes and T2D mellitus as their increased expression has been reported in diabetic conditions.

### cirRNA

CircRNA, a type of closed circular RNA molecule belongs to the ever-growing class of naturally occurring noncoding RNAs (ncRNAs). CircRNAs are covalently closed transcripts that are mostly originated from precursor-mRNA by back-splicing which is a non-canonical event. CircRNAs are mainly generated from exons or introns, by two kinds of splicing, exons reverse splicing of exons and introns [1]. They are evolutionarily conserved, broadly distributed in eukaryotes and highly stable. Some circRNAs are observed to be involved in a variety of functions inside the cell mainly by acting as miRNAs or RNA-binding proteins (RBPs) sponges. CircRNA scan act as a competitive endogenous RNA (ceRNA) by regulating transcription and blocking miRNAs’ inhibition of their target genes. Only a minor fraction of thousands of circRNAs that have been described in human's shows potentially important biological roles [181–183]. A unified explanation for the potential function of the vast majority of circRNAs in disease development is still lacking. CircRNAs are actively involved in the development of many diseases, and their differential expression plays an important role in the disease development. Therefore, decoding the role of lncRNAs and circRNAs in diseases like diabetes and their mechanism of action is an important area in current research. CircRNA-TFRC (transferring receptor, TFRC) is a circRNA that is associated with insulin resistance. It has been observed that over-expression of TFRC can elevate the risk of T2D and metabolic diseases. In initiation and progression of diabetes a number of circRNAs, i.e. circRNA_0054633, circHIPK3, circANKRD36 and circRNA11783-2 have been observed to be involved. Shan et al. [184] found that circ-HIPK3 gets significantly elevated in diabetic retinopathy. Silencing or overexpression of circ-HIPK3 dysregulates the proliferation, migration, viability and tube formation ability of retinal endothelial cells. In study of the molecular mechanism circ-HIPK3, it has been revealed that circ-HIPK3 competitively binds with miR-30a-3p, miR-30d-3p and miR-30e-3p to reverse the expression of their respective target genes, i.e. VEGF, FZD4 and WNT2. This formed a new regulatory network consisting of circRNAs, miRNAs and mRNAs that plays an important role in diabetic retinal vascular dysfunction [185]. A recent study published by Liu et al. (*Proc. Natl. Acad. Sci. U.S.A.* **116**:7455–7464, 2019) demonstrated a novel circRNA cPWWP2A associated with diabetic retinopathy (DR) that can play a role in DR-induced retinal vascular dysfunction through up-regulating the expression of Angiopoietin 1, Occludin and SIRT1 by acting as ceRNA interacting with miR-579. In another study it has been observed that four circRNAs (circCIRBP, circZKSCAN, circRPH3AL and circCAMSAP1) out of the five most abundant in human islets, showed noticeable associations with diabetes status. CircCIRBP showed a role in insulin secretory index in isolated human islets whereas circ CIRBP and circRPH3AL displayed altered expression of elevated fatty acid in treated EndoC-βH1 cells. CircCAMSAP1 was also found to be interfere with T2D status in human peripheral blood. No associations between genotype at T2D risk loci and circRNA expression were identified.

## Discussion

Collective evidence suggests that well-elaborated epigenetic mechanism might be involved in altering the gene expression to influence the etiology of diabetes mellitus and associated complications. Diabetic stimuli activate several epigenetic modifications which may be a key mechanism underlying metabolic memory. Therefore, most of epigenetic factors and mechanism involved in gene modification via upstream signal transduction pathways and other pathways are not known yet. This is the main reason behind the rapid growth and energetic field for identification of new mechanism and therapeutic targets involved in epigenetic changes. Another reason is that the anti-diabetic medication has potential adverse and side effect on the patient such as lactic acidosis, severe hypoglycemia, edema and heart attack and urinary tract infections (Table 1). Several drugs managements have been carried out to controls hyperglycemia and related pathological conditions. Mainly, management of diabetes are limited to two pharmacological interventions such as insulin its related analogous and oral anti-diabetic agents. High blood glucose is frequent and related with extension of complications in diabetes patients. Treatment of hyperglycemia with insulin administration has positive effects on in patients [144,145]. Hypoglycemia is the common side effects of all types of oral anti-diabetic agents. The basal-bolus injection regimens have been used to ameliorate the blood glucose and decrease the diabetes induced complications in patients [146,147]. Hypoglycemia is the common side effects of anti-diabetic therapy and it is main factor for attaining the glycemic control in the inpatient milieu. However, the studies conveyed the relation between hypoglycemia and proliferates the mortality of the patients, but it is still unclear that the hypoglycemia is directly responsible for the mortality of patient or it is only an indicator of the severity of disorder. A study has been shown which underlines beneficial effect of exercise on epigenetic modification in diabetes [148]. Most of the epigenetic changes are reversible in nature, synergetic therapy with epigenetic drugs may be improves the diabetes complication. The several important targets also found in the era of epigenetic modification such as HDAC and DNMT3a. The Set 7 is the inhibitor of histone methyltransferase which is the novel therapy of diabetes mellitus [149]. That helps in the regulation of histone deacetylation and methylation process. Other several important therapeutic targets that are involved in the management of diabetes are worthy of further investigation. Overall, it is expected that further studies in the field of epigenetics will help to identify the new drug biomarkers and drug targets for easy and early management of diabetes and related complications.

**Table 1:** Advantages and disadvantages of existing therapy for diabetes mellitus

Drug type	Advantages	Disadvantages	Generic drug	References
Insulin and its analogs	Adequate lowering blood glucose, antioxidant, anti-inflammatory, anti-platelet and anti-hyperlipidemic activity	Hypoglycemia, need of continuous injection subcutaneously, regular monitoring	Glargine insulin, lispro, aspart and human regular insulin	[144,150–152]
Biguanide	Adequate lowering blood glucose, less chances of hypoglycemia, less cost, used for polycystic ovary syndrome	Risk of Lactic acidosis, heart failure, hepatic cirrhosis and sepsis	Metformin, phenformin	[153–157]
Sulfonylureas and glinides	Adequate lowering blood glucose, oral route	Cardiovascular complications, hypoglycemia	Glibenclamide, Glipizide, repaglinide and nateglinide	[158–160]
Sodium glucose co-transporter inhibitor	Moderate glucose lowering, less hypoglycemia	Glycosuria and UTI	Dapagliflozin Canagliflozin	[161–163]
DPP-4 inhibitor	Moderate glucose lowering, increased the hepatic glucose production	Weight gain and hypoglycemia	Sitagliptin, vildagliptin, sitagliptin and saxagliptin etc.	[164–166]
GLP-1 agonist	Adequate lowering blood glucose and no hypoglycemia	Weight loss, GIT and Pancreatic side effects	Exenatide Liraglutide	[167–170]
α-glucosidase inhibitor	Mild blood glucose lowering, no hypoglycemia and post-prandial hyperglycemia	GIT problems	Acarbose Miglitol	[171,172]

## Abbreviations

AIDS: acquired immune deficiency syndrome
AMPK: AMP-activated kinase
DNA: deoxyribonucleic acid
DNAMT3A: DNA methyltransferase 3α
DNAMT3B: DNA methyltransferase 3β
DPP: dipeptidyl peptidase-4
EOD: early-onset diabetes
GIP: glucose-dependent insulin trophic peptide
GLP: glucagon-like peptide
GLUT4: glucose transporter type 4
GSK3: glycogen synthase kinase 3
GWAS: genome-wide association study
HAT: histone-acetyltransferase
HbA1C: hemoglobin A1c or glycated hemoglobin
HDAC: histone deacetylase
HLA: human leukocyte antigen
HLA-DQA1: HLA class II histocompatibility antigen, DQ α 1
HLA-DRB1: HLA class II histocompatibility antigen, DRB1 β chain
INS: insulin gene
IUGR: intrauterine growth retardation
LYP: lymphoid tyrosine phosphatase
MCP-1: monocyte chemoattractant protein 1
MEF2: myocyte enhancing factor 2
miRNA: microRNA
mRNA: messenger RNA
MS: multiple sclerosis
MyoD: myoblast determination protein
NFATc1: nuclear factor of activated T cell, cytoplasmic 1
OXPHOS: oxidative phosphorylation
PDK4: pyruvate dehydrogenase kinase 4
Pdx1: pancreatic and duodenum homeobox 1
PGC-1α: peroxisome proliferator-activated receptor 1-α
PPARGC1A: peroxisome proliferator-activated receptor γ co-activator 1-α
PTP1B: protein-tyrosine phosphatase 1B
SGLT1: sodium-glucose linked transporter 1
SGLT2: sodium-glucose linked transporter 2
SIRT 1: sirtuin 1
SMARCE 1: SWI/SNF-related matrix-associated actin-dependent regulator of chromatin subfamily E member 1
STAT4: signal transducer and activator of transcription 4
T1D: type 1 diabetes mellitus
T2D: type 2 diabetes
TNFα: tumor necrosis factor α
UCP2: uncoupling protein 2
UHRF: ubiquitin-like, containing PHD and RING finger domains, 1
USF1: upstream transcription factor 1
